# Optimization and development of high-resolution melting curve analysis (HRMA) assay for detection of New Delhi metallo-β-lactamase (NDM) producing *Pseudomonas aeruginosa*

**DOI:** 10.3934/microbiol.2022015

**Published:** 2022-05-09

**Authors:** Sanaz Dehbashi, Hamed Tahmasebi, Mohammad Yousef Alikhani, Fariba Keramat, Mohammad Reza Arabestani

**Affiliations:** 1 Department of Laboratory Sciences, Varastegan Institute of Medical Sciences, Mashhad, Iran; 2 School of Medicine, Shahroud University of Medical Sciences, Shahroud, Iran; 3 Microbiology Department, Faculty of Medicine, Hamadan University of Medical Sciences, Hamadan, Iran; 4 Brucellosis Research Center, Faculty of Medicine, Hamadan University of Medical Sciences, Hamadan, Iran

**Keywords:** *Pseudomonas aeruginosa*, high-resolution melting curve analysis (HRMA), New Delhi metallo-β-lactamase (NDM)

## Abstract

New Delhi metallo-β-lactamase-1 (NDM-1) producing *Pseudomonas aeruginosa* strain detection plays a vital role in confirming bacterial disease diagnosis and following the source of an outbreak for public health. However, the standard method for NDM-1 determination, which relies on the features of the colony of the bacteria cultured from the patient's specimen, is time-consuming and lacks accuracy and sensitivity. This study aimed to standardize a high-resolution melting curve analysis (HRMA) assay to detect NDM producing *P. aeruginosa*. For optimization and development of the HRMA method, a reference strain of *P. aeruginosa* was used. For evaluating the broad range PCR data, ABI Step One-Plus Manager Software version 3.2 and Precision Melt Analysis Software 3.02 (Applied Biosystems) were used.

Based on the results, expected results were obtained for all tested strains, with high analytical sensitivity and specificity. Temperature melting analyses of the HRMA time PCR assays showed the Tm at 89.57 °C, 76.92 °C and 82.97 °C for N-1, N-2 and N-3 genes, respectively. Also, melting point temperatures of the *bla*_VIM_, *bla*_SPM_ and *bla*_SIM_ amplicons for isolates identified as MBL strains were 84.56 °C, 85.35 °C and 86.62 °C, respectively. The amplification results using negative control genomes as templates were negative, showing the specificity of the designed assays. Our study's data indicated that the sensitivity and specificity of the HRMA method are linked to the primer length and the fluorescent dye. We can further identify antibiotic resistance in NDMproducing *P. aeruginosa* by software analysis and melting curve analysis.

## 1. Introduction

*Pseudomonas aeruginosa* is a significant microorganism involved in urinary, bloodstream, pulmonary, soft tissue and surgical site infections [1],[2]. Antibiotic resistance is one of the most pressing problems in public health and will remain threatening to modern medicine in the coming decades. Because of the increasing abuse of antibiotics in hospitals and the community, some widely used antibiotics are losing their function, and scientists and doctors must develop new antibiotics to overcome this problem. [3],[4]. Metallo-β-lactamaseproducing (MBL) strains to hydrolyze the β-lactam ring of the drug compound, thereby inactivating them. In contrast to serine β-lactamases, MBLs use at least one but more commonly two Zn^2+^ ions in their active site to catalyze the hydrolysis of β-lactam rings [5],[6]. There are various methods for identifying MBL and NDM-producing strains, which fall into two phenotypic and genotypic groups. Usually, phenotypic methods have low specificity and low speed, and error results [7],[8]. Therefore, it is necessary to use molecular methods along with phenotypic methods. High-resolution melting (HRMA) analysis is one of the most sensitive and precise molecular methods based on real-time PCR [9],[10].

HRMA is used to characterize bacterial DNA samples according to their dissociation behavior as they transition from double-strand DNA to single-strand DNA with increasing temperature and fluorescence detection [8],[11]. The HRMA is entirely precise warming of the amplicon DNA from around 50 °C up to around 95 °C. During this process, the amplicon's melting temperature is reached, and the two strands of DNA separate or “melt” apart. The concept of HRMA is to monitor this process happening with real-time PCR [12]. This rationale is achieved by using intercalating dyes. The intercalating dye binds explicitly to double-strand DNA and forms the stable fluorescent, and thus one can monitor the relative quantity of the product during DNA amplification in real-time PCR [13]. However, HRMA takes it one step further in its ability to capture much more detail. It has an increased resolving power, as melting curves from different amplicons may be differentiated based on the melt curve's shape even when the melt temperature values are the same [14],[15].

HRMA has the potential to be a powerful tool in the clinical microbiology laboratory, providing rapid detection of genetic determinants conferring antibiotic resistance to complement current phenotypic antimicrobial susceptibility testing methods [16],[17]. These methods are labor-intensive, expensive and require unique expertise, and the results are difficult to interpret [18]. An accurate, rapid and cost-effective typing scheme is urgently needed for active surveillance and epidemiological investigations [19]. HRMA typing is a compassionate, rapid and cost-effective option for detection purposes [20],[21]. It can perform highly accurate genotyping to a hefty quantity of samples in a short amount of time [8],[22]. HRMA also is a straightforward technology that can perform both the PCR analysis and HRMA in one instrument [20]. This reduces extra expenses, saves time and creates a simpler workflow [16]. It is only necessary to create the PCR reaction volume for each sample to be analyzed, and it eliminates the need for solvents and electrophoresis gels [22].

Therefore, we try to extend the application of the HRMA assay to this area to help detect the antibiotic resistance in different strains of NDM producing *P. aeruginosa*. We modified the multiplex HRMA to amplify both the bacteria NDM producing gene and MBL producing gene and analyze its melting curve.

## 2. Materials and methods

### 2.1. Subcultures of the reference strain

Subcultures of *P. aeruginosa* ATCC 15442, *P. aeruginosa* PAO-1, and *P. aeruginosa* ATCC 27853 were used from Hamadan Medical University, Microbiology Department microbial bank. Reference strains were cultivated at 37 °C for 24 h in cetrimide agar (Merck, Germany). All strains were kept at −20 °C in TSB containing 20% glycerol. The Ethics Committee approved this study of Hamadan University of Medical Sciences (Code No: IR.UMSHA.REC.1398.573).

### 2.2. DNA extraction and Sanger sequencing

*P. aeruginosa* DNA extraction was performed using a DNA extraction kit (Qiagen, Germany); the steps were followed according to the kit protocol. DNA concentration was determined using a spectrophotometer (Nanodrop-200, Hangzhou Allsheng Instruments Co., Ltd., China). In this study, the sequencing method used was the dideoxy chain-terminating Sanger method [23].

### 2.3. Real-time PCR and primer sensitivity and specificity

Primer sequences were initially set up as outlined in previous studies [24]–[27] (Table 1). For sensitivity and specificity of primers, nine-fold serial dilutions of 0.5 McFarland DNA (1.5 × 10^8^ CFU/mL) were made (1:1–1, 1:1–2, 1:1–3, 1:1–4, 1:1–5, 1:1–6, 1:1–7, 1:1–8). Serial dilution real-time PCR tested primers' efficiencies for both target genes and reference genes. Standard curves were constructed by the Ct (y-axis) versus log DNA dilution (x-axis). The primer efficiency (*E*) of one cycle in the exponential phase was calculated according to the equation: *E* = 10−^(1/slope)−1^ × 100 [28].

Briefly, 2 µL (0.5 µM) of each primer, 2 µL of DNA template, 4 µL of EvaGreen, and made up to a final volume of 20 µL using ddH_2_O. Real-time PCR reactions were performed on the ABI real-time machine (ABI StepOnePlus, USA). The thermal cycles were set for reverse transcription steps (55 °C for 5 min, 95 °C for 10 min, 95 °C for 20 s) followed by PCR steps: 95 °C for 15 s, 59 °C for 30 s, repeated for 40 cycles. The slope value was calculated from serial dilutions for each gene, which was then used to determine the reaction's efficiency [12].

**Table 1.** Oligonucleotide sequences used in this study.

Target	Primer Name	Sequence of Primers	Melting Tm (±0.5 °C)	Accession Number	Primer Location	Product Size (bp)	Ref
NDM-1	*N-1*	F: GACCGCCCAGATCCTCAA R: CGCGACCGGCAGGTT	89.57	MN193055.1	71–122	55	[24]
	*N-2*	F: TTGGCCTTGCTGTCCTTG R: ACACCAGTGACAATATCACCG	76.92	MH168506.2	630–586	85	[25]
	*N-3*	F: GCGCAACACAGCCTGACTTT R: CAGCCACCAAAAGCGATGTC	82.97	MK371546.1	298–452	155	[23]

MBL	*bla*_SIM_	F: TACAAGGGATTCGGCATCG R: TAATGGCCTGTTCCCATGTG	85.35	KX452682.1	1–570	570	[27]
	*bla*_VIM_	F: TCTCCACGCACTTTCATGAC R: GTGGGAATCTCGTTCCCCTC	84.56	NG_068039.1	332–455	124	[26]
	*bla*_SPM_	F: AAAATCTGGGTACGCAAACG R: ACATTATCCGCTGGAACAGG	86.62	KX452683.1	1–271	271	[27]

### 2.4. Evaluation of sensitivity and specificity of HRMA assay

The efficiency and the analytical sensitivity of the HRMA-PCR were evaluated by triplicate testing of 9-fold serial dilution series of each of the three reference strains. The Applied Biosystems StepOnePlus real-time PCR system was used to amplify and detect products. The reaction mix was prepared using the following components for each of the samples: 4 µL of Master Mix HRMA (HOT FIREPol EvaGreen HRMA Mix), 1 µM of each respective primer and 12 µL of DMSO (Sigma-Aldrich, USA). The following cycle parameters were used: 2 min at 50 °C, 10 min at 95 °C. Moreover, 40 cycles with denaturing for 15 s at 95 °C and by annealing/elongation for 1 min at 60 °C. Melting curves were generated after each run to confirm a single PCR product (from 60 °C to 95 °C, increasing 1 °C/3 s).

### 2.5. Data analysis

Data analysis was performed using ABI Thermo Fisher software (release 2018, version 3.0.2) and BioEdit 7.4 software (Caredata, Inc., USA). Normalized and difference plots were generated.

## 3. Results

### 3.1. Analytical sensitivity and specificity of primers

**Figure 1.**
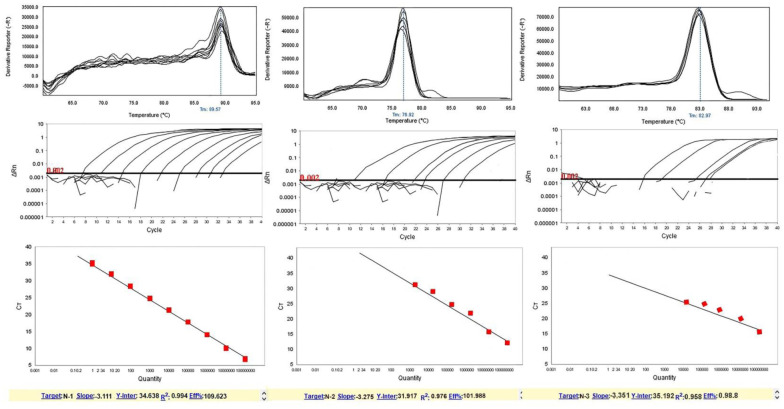
Analytical sensitivity of real-time PCR and examples of optimization of primer pairs based on melting curve analysis for NDM primers to detect NDM producing *P. aeruginosa* strains. The melting curves for each primer pair were investigated: (left) N-1 gene with a melting point of 89.53 ± 0.5 °C, (middle) N-2 gene with a melting point of 76.92 ± 0.5 °C, (right) N-3 gene with a melting point 82.97 ± 0.5 °C. The mean of a: 10^8^; b: 10^7^; c: 10^6^; d: 10^5^; e: 10^4^; f: 10^3^; g: 10^2^; h: 10^1^ and i: 10^0^ CFU/mL of DNA dilutions. Bold black horizontal lines represent the cycle threshold of real-time PCR. One peak with a shoulder corresponds to genomic DNA amplification; no peak corresponds to no amplification. EvaGreen color and single-tube reactions were used in this test. Also, real-time PCR was performed as a single step.

**Figure 2.**
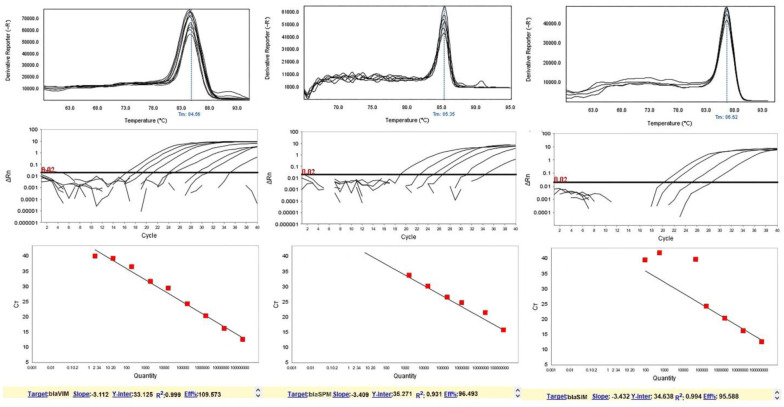
Analytical sensitivity of real-time PCR and examples of optimization of primer pairs based on melting curve analysis for MBL primers to detect NDM producing *P. aeruginosa* strains. The melting curves for each primer pair were investigated: (left) bla_VIM_ gene with a melting point of 84.56 ± 0.5 °C, (middle) bla_SPM_ gene with a melting point of 85.35 ± 0.5 °C, (right) blaSIM gene a melting point of 86.62 ± 0.5 °C. The mean of a: 10^8^; b: 10^7^; c: 10^6^; d: 10^5^; e: 10^4^; f: 10^3^; g: 10^2^; h: 10^1^ and i: 10^0^ CFU/mL of DNA dilutions. Bold black horizontal lines represent the cycle threshold of real-time PCR. One peak with a shoulder corresponds to genomic DNA amplification; no peak corresponds to no amplification. EvaGreen color and single-tube reactions were used in this test. Also, real-time PCR was performed as a single step.

**Figure 3.**
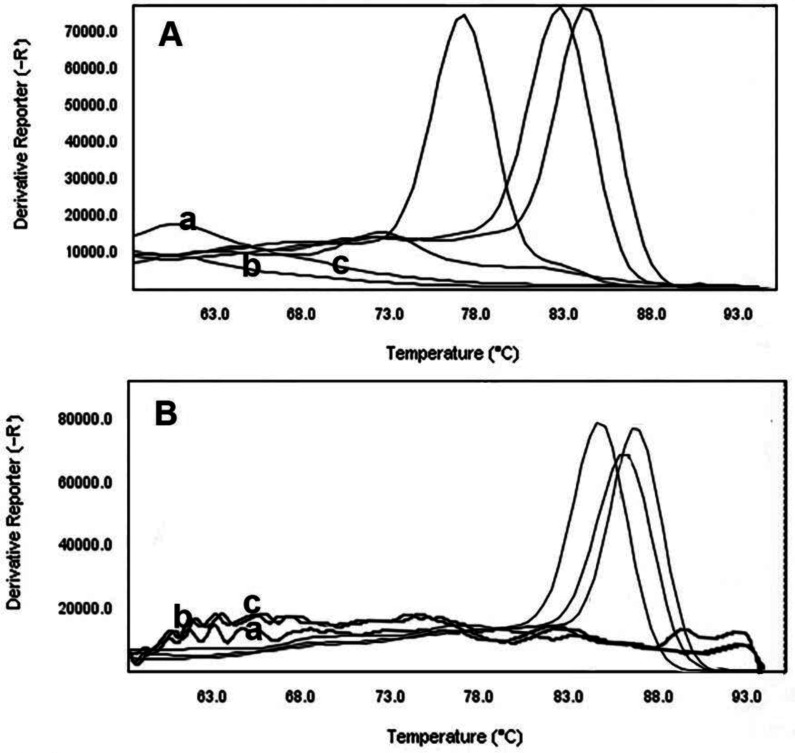
Melting curve analysis and analytical specificity of real-time PCR for NDM primers (A) and MBL primers (B) used to detect NDM producing *P. aeruginosa* strains: (a) blank tube, (b) *P. aeruginosa PAO-1* and (c) *P. aeruginosa* ATCC 27853. One peak with a shoulder corresponds to genomic DNA amplification; no peak corresponds to no amplification. EvaGreen color and single-tube reactions were used in this test. Also, real-time PCR was performed as a single step. A 0.5-McFarland concentration (1.5 x 10^8^ CFU/mL of DNA) was used to determine primer specificity.

After 9-fold dilutions, a high CT was observed in the 10^0^ CFU/mL and low CT in the 10^8^ CFU/mL. Moreover, increasing CT values were identified as the inhibitor binding to the DNA, as such binding will reduce the amount of template available for amplification. More comprehensive CT value ranges indicated more binding; likewise, smaller ranges corresponded to less interaction between DNA and inhibitor. The CT values for these cell densities were within the 9 to 40 cycle range in the amplification process, while higher DNA concentrations appeared within 9 to 31 cycles. As seen in Figures 1 and 2, relative to a linear range of each standard curve, melting peaks can be seen for many lower DNA concentrations; however, the concentrations could not be quantified. Therefore, the actual quantitative, linear portions of the calibration curves did not extend as low as the detection limit. Melting curves displayed a single melting Tm: 89.57 °C for the N-1 gene, 76.92 °C for the N-2 gene, 82.97 °C for the N-3 gene, 84.56 °C for the *bla*_VIM_ gene, 86.62 °C for the *bla*_SIM_ gene, and 85.35 °C for the *bla*_SPM_ gene (Figures 1 and 2). Samples containing DNA exhibited positive real-time PCR amplification, and negative controls failed to show amplification. Also, serial dilutions of positive-control DNA amplification curves showed Ct values inversely related to template DNA concentration (Figure 3).

Reaction efficiencies were found to be within the range of 3 to 3.5 when calculated from the standard curves using the ABI Thermo Fisher analysis software (Version 2.3.2) with a formula of *E* = 10^(−1/slope)^−1. For the N-1, N-2 and *bla*_VIM_ primer set, the reaction efficiency reached a value slightly greater than 3, at 3.2, which would suggest the efficiency of 101%. Efficiencies greater than 100% can be obtained. All the investigated dilutions showed low efficiencies: N-3, *E* = 98.8%; *bla*_SMP_, *E* = 95.588%; *bla*_SIM_, *E* = 96.493 (Figures 1 and 2).

For the N-1 and *bla*_VIM_ primer set, the linear range was determined to extend as low as 10^0^ CFU/mL, N-2 was 10^3^ CFU/mL, N-3 was 10^4^ CFU/mL, *bla*_SMP_ was 10^2^ CFU/mL, and *bla*_SIM_ was 10^5^ CFU/mL, as indicated by the lowest DNA concentration value on each of the standard curves. Points that caused the curves to deviate from linearity (mostly those with lower concentrations) were excluded (Figures 1 and 2).

### 3.2. Sensitivity and specificity of HRMA assay

**Figure 4.**
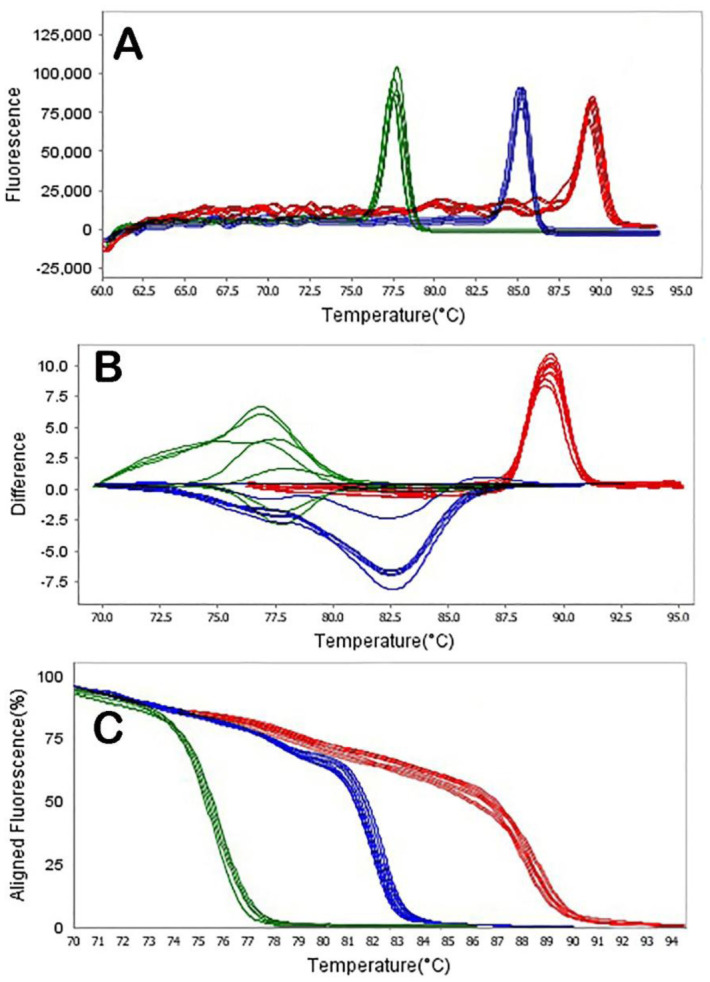
HRMA graphs corresponding to one high-resolution melting analysis of a subset of NDM producing *P. aeruginosa* strains by *N*-1 (A), *N*-2 (B) and *N*-3 (C) genes. DNA samples from all the dilutions involved in this study were prepared and amplified successfully using the EvaGreen dye-based method in the ABI instrument. Primers' specific melting peaks (Tm) were obtained via HRMA, allowing the differentiation of all investigated β-lactamase enzymes. Due to the positively saturating EvaGreen dye and the HRMA, the resolution accuracy was ±0.1–0.5 °C.

**Figure 5.**
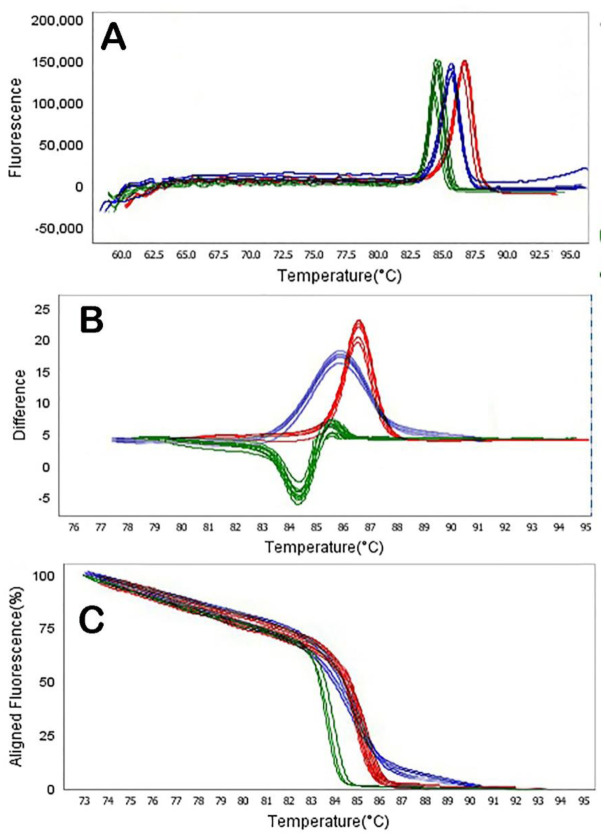
HRMA graphs corresponding to one high-resolution melting analysis of a subset of NDM producing *P. aeruginosa* strain by *blaVIM* (A), *blaSIM* (B) and *blaSPM* (C) genes. DNA samples from all the dilutions involved in this study were prepared and amplified successfully using the EvaGreen dye-based method in the ABI instrument. Primers' specific melting peaks (Tm) were obtained via HRMA, allowing the differentiation of all investigated β-lactamase enzymes. Due to the positively saturating EvaGreen dye and the HRMA, the resolution accuracy was ±0.1 °C–0.5 °C.

Fluorescence data were analyzed using the tools for HRMA incorporated in the ABI Thermo Fisher analysis software. HRMA PCR amplification curves of samples analyzed for the presence of NDM producing *P. aeruginosa* are shown in Figures 3, 4, and 5. Difference plots of normalized data show the differences in fluorescence between each sample of DNA. Derivative plots display the rate of fluorescence change; the peak indicates the melting temperature of a sample. All plots displayed a single melting domain, typically between 87.07 °C–87.57 °C for the N-1 gene, 76.42 °C–76.92 °C for the N-2 gene, 82.47 °C–82.97 °C for the N-3 gene, 84.06 °C–84.56 °C for the *bla*_VIM_ gene, 86.12 °C–86.62 °C for the *bla*_SIM_ gene and 85.30 °C–85.80 °C for the *bla*_SPM_ gene, following different product sizes.

The results of this representative experiment show that all samples containing *P. aeruginosa* DNA had measurable amplification, as detected by exponential fluorescence (Figure 3), and all the DNA dilutions of NDM producing *P. aeruginosa* were identified (dilution of 10^8^ to 10^0^ CFU/mL). The N-1 and *bla*_VIM_ genes in NDM producing *P. aeruginosa* were detected in all dilutions of DNA. Moreover, N-2, N-3, *bla*_SPM_ and *bla*_SIM_ primers can be able to detect bacterial DNA in dilutions of 10^3^ CFU/mL, 10^4^ CFU/mL, 10^2^ CFU/mL and 10^5^ CFU/mL, respectively (Figures 4 and 5).

The software automatically analyzed the raw melting curve data and set the starting (pre-melt) and ending (post-melt) fluorescence signals of all data to actual values to aid interpretation and analysis (Figures 4 and 5). The cursors for these two points have defaulted to the ends of the curve. However, these regions were manually adjusted to encompass a representative baseline for the pre-melt and post-melt phases. Widening the normalization regions into the melt phase was avoided to ensure that curves normalize effectively. Moreover, we performed a melt curve analysis of HRMA PCR samples to assess the amplicon's specificity. The results of the HRMA showed a very similar melt peak for all serial dilutions of *P. aeruginosa*.

## 4. Discussion

Based on the current study, the efficiency of genes was at least 99.99%, the r2 was >0.99.99, and melt curves yielded single peaks. Interestingly, the Ct vs. DNA relationship's slope varied little across the nine-fold dilutions tested, ranging from −3.589 to −3.955. According to previous studies, this can be justified because short fragments bind less fluorescent and are compensated by a higher primer concentration [29],[30]. However, sometimes the peak height of the short amplicon increases in a different replicate. This problem gets worse in the MCA, such that sometimes we lost the long amplicon even at the primer ratio of 1:1. Furthermore, these results agree with Mentasti et al. [5].

Moreover, three *bla*NDM-1 primers with different amplicon length and MBL primers were used to detect NDM producing *P. aeruginosa* strains. These results indicated that the primers' specificity, with a 0.5 °C error range, could detect NDM producing *P. aeruginosa*. Andini et al. showed that an accurate analysis of the melting curve could play a significant role in the diagnosis [31]. Ashrafi et al. found that, to obtain the best performance in sophisticated methods such as HRMA, the DNA's melting temperature must be monitored in various dilutions to obtain accurate sensitivity and specificity [21]. Tahmasebi et al. also confirmed that efficiency is probably due to the shorter length of primers' products, which enabled better amplification in PCR [9].

Based on the current study, Figures 1 and 2 showed that the highest analytical sensitivity and specificity were reported for the detection of antibiotic-resistant strains, as indicated by the lowest DNA concentration value on each of the standard curves. Smiljanic et al. also illustrated that identifying Gram-negative NDM and MBL producing strains is difficult because the resistance to carbapenems in these bacteria is encoded by similar sequences [32]. Thus, using a sensitive and precise method such as HRMA and specific primers could identify strains such as MBL and NDM producing strains. In a study, Ding et al. proposed the PASGNDM699 strain resistance to a wide range of antibiotics. They also confirmed the clinical importance of PASGNDM strains in causing resistant infections [33].

Based on Figure 4 and Figure 5, DNA dilutions of NDM producing *P. aeruginosa* strains were identified (dilution of 10^8^ to 10^0^ CFU/mL). The results were different from those obtained by Naas et al. and Smiljanic et al. [32],[34]. Identification of MBL and NDM producing strains has been performed in various studies in Sweden [17], USA [35], Australia [25] and Italy [36] in Gram-negative bacteria by the HRMA method.

Nevertheless, one of the most important benefits of the multiplex HRMA method is the simultaneous identification of different NDM varieties. Identification of these variants using phenotypic methods has low accuracy and speed. Those methods also require spending much time optimizing. Makena et al. found that the identification of NDM variants by phenotypic methods requires protein stability and is not practical due to the evolution of NDM-producing strains [37].

According to our results, primers with a short length had the best sensitivity and specificity in the HRMA assay. Słomka et al. demonstrated that when the DNA quality is low, DNA degradation or long DNA breaks during extraction makes the long template harder to amplify [20]. Though, the capacity to monitor PCRs in real-time has revolutionized how PCR is used in the clinical microbiology field. HRMA assay is used to amplify and concurrently quantify a targeted DNA molecule and enables both detection and quantification of DNA. HRMA PCR needs a fluorescent reporter that binds to the finished product and reports its presence by fluorescence. The Eischeid study confirms these results [38].

In this study, we optimized the HRMA method to identify antibiotic resistance gene variants. We did not use designed primers in this study. The design and optimization of C + G values ​​ in designed primers provide the sensitivity and specificity of HRMA in the simultaneous identification of drug resistance [39]. Thus, the length of the selected primers should be considered to identify bacterial sub-strains. Another limitation of our study was the lack of use of different fluorescent dyes in identifying subtypes. Based on previous studies, the type of dye used significantly affects the sensitivity and specificity of HRMA [40]. Therefore, in different master mixes from different suppliers, calibration is necessary to establish the new Tm data on the reference and clinical strains.

## 5. Conclusion

We demonstrated that the HRMA assay is a rapid and sensitive pre-sequence screening tool that allows the detection of low DNA concentration. Further, our study results showed that NDM and MBL genes' co-existence could be detected using the HRMA method reference strains. Moreover, the HRMA method for identifying NDM and MBL producing strains has high sensitivity and specificity. The present study also confirmed that primer product length and fluorescent dye play critical roles in increasing the sensitivity and specificity of the HRMA assay. However, the selection of the melting temperature range is essential to the analysis, as there needs to be sufficient data both before and following the melting transition to allow reliable normalization of the melting curves.
